# Stridor in a Newborn with Double Aortic Arch-A Case Report

**Published:** 2019-01

**Authors:** Elsie-Jane Anastasius, Halimuddin Sawali

**Affiliations:** 1 *Department of Otorhinolaryngology, Hospital Queen Elizabeth, Kota Kinabalu, Sabah, Malaysia. *

**Keywords:** Double aortic arch, Stridor, Vascular ring

## Abstract

**Introduction:**

Double aortic arch (DAA) is a congenital anomaly of the aortic arch. It is the most common type of complete vascular ring. When it occurs, the connected segment of the aortic arch and its branches encircle the trachea and esophagus, leading to symptoms related to these two structures.

**Case Report:**

We present a case of a newborn baby who developed biphasic stridor immediately after a normal vaginal delivery. Endoscopic assessment of the trachea revealed a pulsatile narrowing at the level of the thoracic trachea, suggestive of an external compression. A contrast-enhanced computed tomography scan of the thorax with three-dimensional reconstruction confirmed the diagnosis of DAA with compression of the trachea and esophagus.

**Conclusion:**

Clinicians should strongly consider the possibility of a congenital vascular ring compression should an infant with a normal upper airway present with stridor. A precise diagnosis can be made by radiological examination.

## Introduction

Double aortic arch (DAA) is the most common type of complete vascular ring that encircles the trachea and esophagus completely by the connected segment of the aortic arch and its branches(1), often resulting in variable airway compression.

## Case Report

A 31-year-old woman gave birth to a full-term baby girl weighing 3.6 kg via spontaneous vaginal delivery at 40 weeks of gestation. The Apgar score was 9 after 1 minute; however, the child was noted to have a loud biphasic stridor with subcostal recessions, which did not improve with positioning. The respiratory rate was 60 breaths per minute and the pulse oximeter oxygen saturation was 100% under a head box with oxygen (5L/min). Liquor was clear. A physical examination of the cardiorespiratory system revealed normal heart sounds with transmitted sounds heard on auscultation of the lung fields. The case was referred to the otorhinolaryngology team on day 1 of life after being transferred from a district hospital. A bedside flexible nasolaryngoscopy examination revealed normal glottic and supraglottic structures. 

**Fig 1:**
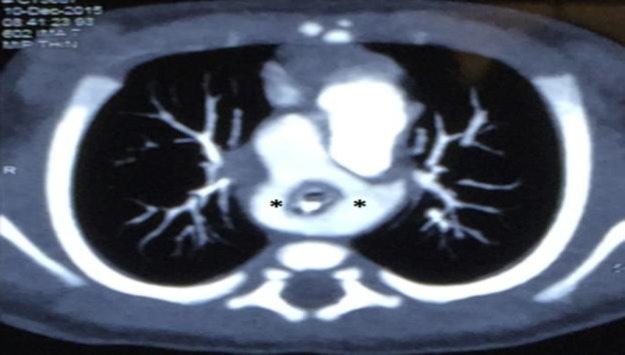
Contrast-enhanced computed tomography (CECT) of the thorax showed both aortic arches form a complete vascular ring, which encasing the trachea and esophagus. Asterisk (*), complete vascular ring

**Fig2:**
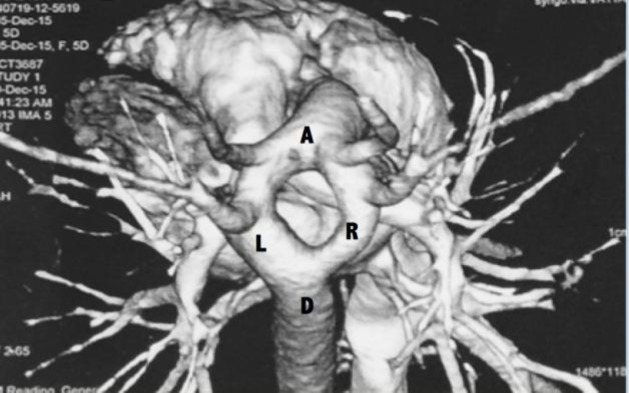
Computed tomogram (3D reconstruction) of the arch of the aorta showing a complete vascular ring. A, ascending aorta; D, descending aorta; L/R, left and right arch

A direct laryngoscopy with telebronchoscopy carried out on day 2 of life revealed a pulsatile narrowing of the tracheal lumen at the level of the thoracic trachea, suggestive of an external compression. An uncuffed endotracheal tube with an internal diameter of 3.0 mm was used to stent the narrowed segment to maintain airway patency. A contrast-enhanced computed tomography (CECT) of the thorax with a three-dimensional (3D) reconstruction confirmed a diagnosis of DAA, which was tightly encasing the trachea and esophagus (Fig.1,2).

Echocardiography did not show any associated intracardiac anomaly. Feeding was maintained via a nasogastric tube from day 1 of life. Although corrective surgery was scheduled at a cardiac center in Kuala Lumpur, the infant developed acute massive hematemesis on day 37 of life and succumbed to it prior to the surgery.

## Discussion

Vascular rings are rare congenital vascular disorders that inevitably encircle the trachea and esophagus. DAA is the most common form of complete vascular ring malformation (1), and is due to the failure of regression of the right fourth arch during embryonic aorta development (2). In development, the remnant of the right fourth arch will later become the right innominate artery, and the left fourth arch becomes the aortic arch.

Respiratory symptoms at birth should raise the suspicion of vascular ring compression, especially in patients with normal fiberoptic findings of the larynx and also those exhibiting signs and symptoms of laryngomalacia. Patients may present with stridor, persistent cough, wheezing, and recurrent respiratory infection during infancy (3). Gastrointestinal symptoms such as feeding difficulties and vomiting are the common associated symptoms (4).

In this case, the child presented with biphasic stridor at birth. Direct laryngoscopy with telebronchoscopy under general anesthesia revealed a pulsatile narrowing of the tracheal lumen at the level of the thoracic trachea. 

A CECT with 3D reconstruction revealed the anomalous construction of the aortic arch. As in this case, CECT is one of the imaging techniques of choice for obtaining a diagnosis and when planning for surgical intervention. Early surgical intervention, by dividing the arches and freeing the trachea and esophagus from the surrounding tissue, is of the utmost importance to prevent late complications, such as tracheomalacia or aortoesophageal fistula (4,5). The risk of aortoesophageal fistulas can be further reduced by avoiding prolonged nasogastric tube intubations (5). 

As the child developed sudden massive hematemesis, the possibility of an aortoesophageal fistula cannot be ruled out in this case.

## Conclusion

In conclusion, clinicians should strongly consider the possibility of a congenital vascular ring compression should an infant with a normal upper airway present with stridor. A precise diagnosis can be made by radiological examination.
